# Heterogeneous phenotype and cardiovascular comorbidities in Swedish patients with spinobulbar muscular atrophy

**DOI:** 10.1007/s00415-025-13605-z

**Published:** 2026-01-10

**Authors:** Anna-Karin Roos, Simon Forsberg, Erica Stenvall, Peter M. Andersen, Per Zetterström, Angelica Nordin, Karin M. E. Forsberg

**Affiliations:** 1 Department of Clinical Sciences, Neurosciences, Umeå University, 90184 Umeå, Sweden; 2 Department of Medical Biosciences, Umeå University, Umeå, Sweden

**Keywords:** Spinobulbar muscular atrophy, Motor neuron disease, Phenotype, Cardiovascular disease, Neurofilament light chain

## Abstract

**Background:**

Spinobulbar muscular atrophy (SBMA) is an X-linked neuromuscular disorder characterized by adult-onset progressive muscle atrophy, flaccid paresis, and bulbar palsy. In addition, increasing evidence indicates that SBMA is a multisystem disorder with prominent non-motor symptoms, such as sensory neuropathy, androgen insensitivity, and glucose intolerance. This study aimed to further characterize the clinical manifestations and biomarker profile in a large Swedish SBMA cohort.

**Methods:**

49 genetically confirmed SBMA patients were identified from a motor neuron disease database at Umeå University Hospital, Sweden. CAG repeat length in the androgen receptor (*AR)* gene was assessed by RP-PCR. Blood samples were analyzed for cardiovascular and muscle biomarkers. Clinical data were collected from medical records and interviews, with autopsy findings reviewed in two cases.

**Results:**

The mean CAG repeat length was 43.1, with a mean age at motor symptom onset of 58.6 years. Notably, 19% of patients initially presented with sensory symptoms. High prevalence of hypertonia (70%), diabetes mellitus (39%), and cardiac disease (38%) was observed. Elevated troponin levels were common, and pNfL (neurofilament light chain in plasma) was elevated in seven patients, likely reflecting combined cerebrovascular and cardiovascular comorbidity. Importantly, two of these seven patients exhibited rapid disease progression, and a concomitant diagnosis of ALS was confirmed histopathologically.

**Discussion:**

This cohort was characterized by a relatively low number of *AR* gene CAG repeats and a late onset of motor symptoms. Sensory symptoms frequently occurred before motor decline. Cardiovascular disease and diabetes were common comorbidities and, in some cases, preceded neurological symptoms. These findings underscore the need for improved clinical awareness of the heterogeneous presentation of SBMA and support routine cardiovascular monitoring to reduce diagnostic delays and prevent early mortality.

**Supplementary Information:**

The online version contains supplementary material available at 10.1007/s00415-025-13605-z.

## Introduction

Spinobulbar muscular atrophy (SBMA), caused by a coding CAG nucleotide repeat expansion in the *androgen receptor (AR)* gene, is an X-linked recessive adult-onset progressive neuromuscular disorder with an estimated prevalence of approximately 3 per 100,000 males [1]. Traditionally, SBMA is clinically characterized by muscle twitching (fasciculation), tremors, progressive flaccid paresis, and atrophy affecting both proximal and distal limb muscles, as well as muscles innervated by bulbar nerves [2].

In addition to motor symptoms, non-motor features of SBMA—namely, sensory neuropathy, impaired glucose tolerance, hyperlipidemia, autonomic dysfunction, and cardiac arrhythmias—have been reported [2]. AR loss of function underlies the partial androgen insensitivity observed in SBMA, contributing to clinical features, such as gynecomastia and reduced fertility [3]. In contrast, the motor and sensory manifestations are attributed to a toxic gain-of-function mechanism, where androgen-dependent accumulation of the mutant AR protein results in the formation of intra-nuclear and cytoplasmic inclusions. Such pathological inclusions have been identified not only in motor neurons [4] but also in non-neuronal tissues and visceral organs [5]. The precise molecular mechanisms underlying the loss-of-function and gain-of-function manifestations of SBMA remain incompletely understood.

Here, we investigated a cohort of 49 individuals with SBMA, primarily from northern Sweden. We characterized each participant’s clinical phenotype and number of CAG repeats and evaluated biomarkers associated with both neuromuscular and cardiovascular function. Notably, we found a high prevalence of cardiovascular diseases, diabetes mellitus, and late-onset muscular symptoms, highlighting the need for clinicians to be aware of the broad spectrum of other diseases associated with SBMA both when diagnosing patients with SBMA and when managing the disease in the clinic.

## Methods

### Participants

Patients with different types of motor neuron diseases (MNDs) were recruited to participate in molecular biology and genetics research at Umeå University Hospital in northern Sweden starting in 1992. We selected all individuals with a diagnosis of SBMA from our database until 2025. Individuals without a Swedish social security number were excluded (5 individuals originating from other countries). The final cohort consisted of 49 individuals with symptomatic SBMA.

### Data collection

Clinical information on age at onset, initial symptoms, and concomitant diseases was obtained from medical records and, when possible, through contact with patients and relatives. Data collection was cross-sectional. For most of the deceased individuals, a larger part of the course of disease could be reconstructed. For three individuals, all clinical data were missing, and some deceased patients lacked clinical data on comorbidities. At the time of recruitment, peripheral blood was collected by standard venipuncture in EDTA-containing tubes, and after centrifugation, plasma, buffy coat, and red blood cells were placed in separate tubes and stored at − 80 °C in the Neurobiobank at Umeå University Hospital. From the tubes containing the buffy coat, DNA was extracted for genetic analysis using NUCLEON BACC3 (Cytiva, Global Life Sciences Solutions, Buckinghamshire, UK) according to the manufacturer’s recommendations. In some individuals, blood samples were collected repeatedly during the disease course. Blood for biomarker studies was unavailable from four individuals (Supplemental Table S1, Nos. 7, 24, 32 and 39) and was missing for a single biomarker in two individuals (Supplemental Table S1, Nos. 47 (TnI) and 48 (ProBNP)).

### Repeat expansion analysis

The *AR* gene expansion size was determined using fragment length analysis. Amplification of the DNA fragment was performed using OneTaq (New England Biolabs) according to the manufacturer’s recommendations (for primer sequences and PCR conditions, see online Supplemental Tables S2 and S3). The samples were analyzed using a 3500XL Genetic Analyzer (Applied Biosystems, Life Technologies, Singapore) and the software Peak scanner version 3.0. Alleles with fewer than 36 repeats were defined as nonpathogenic, and those with 38 repeats or more were defined as pathological [6]. Alleles with 36–37 repeats were defined as pathogenic with reduced penetrance. All individuals were also screened for a hexa-nucleotide repeat expansion in *C9ORF72* and for pathogenic variants of *SOD1* as described previously [7,8].

### Biochemistry

Standard laboratory analyses were performed according to validated, accredited clinical practice at the Clinical Laboratory at Umeå University Hospital. Plasma samples were analyzed on a Cobas Pro 6000 (Roche Diagnostics, Rotkreuz, Switzerland) for the following biomarkers: creatine kinase (CK), creatinine, high-sensitivity cardiac troponin T (TnT), myoglobin and, pro-B-type natriuretic peptide (ProBNP)**.** HbA1c was analyzed on a TOSOH G8LA, (Tosoh Corporation, Tokyo, Japan). High-sensitivity cardiac troponin I (TnI) was analyzed at the Sahlgrenska University Hospital laboratory using the Alinity i platform, (Abbott Diagnostics, Lake County, IL, USA). Levels of plasma neurofilament light chain (pNfL) were analyzed in duplicate by single-molecule array (Simoa) technology using a digital immunoassay Simoa HD-X Analyzer (Quanterix Billerica, MA, USA) with the NF-Light v2 Advantage Kit at Umeå University Hospital. The intra- and interplate coefficients of variation (CV% values) were < 7 and < 14, respectively. We used the age-dependent reference interval established by Simrén et al. [9].

### Immunohistochemistry

Tissues were collected postmortem from the cervical, thoracic, and lumbar regions of the spinal cord; the frontal, motor, and parietal cortex; and the brainstem at the level of the hypoglossal nucleus. The tissues were fixed by immersion in 4% paraformaldehyde in 0.1 M Na phosphate, pH 7.4, at room temperature. Paraffin-embedded Sects. (4 μm) were stained with hematoxylin and eosin. Immuno-histochemical staining was performed using antibodies against the following: polyQ (MAB1574, Chemicon, dilution 1:450), p62 (610,833, BD Transduction Laboratories, dilution 1:50), pTDP-43 (CAC-TIP-PTD-M01A, Cosmo Bio Ltd, dilution 1:3000), and poly-DPR-GR (MABN778, Sigma Aldrich, dilution 1:2000). For immunohistochemistry, staining was performed according to the manufacturer’s recommendations using a BenchMark Ultra (Ventana Medical Systems, Roche Group, Tucson, AZ, USA) with an UltraView detection kit. The sections were counterstained with hematoxylin, washed, and mounted with Glycergel Mounting Medium (DakoCytomation). Two independent raters (K.F. and E.S.) performed the neuropathological assessments.

### Statistical analysis

Chi-square test was used to evaluate the association between CAG repeat length, sensory-onset, and cardiac disease. P values were calculated using R studio software (version 2025.09.1 + 401).

### Standard protocol approvals, registrations, and patient consent

The study was performed in accordance with the 1964 Declaration of Helsinki with later amendments and was approved by the Swedish Ethical Review Authority (FEK 94–135, with later amendments, most recently EPN 2018-496-32 M and EPN 2014-17-31 M, for histopathological studies). Written informed consent was obtained from all participants, and the consent included permission to publish and present the scientific results.

### Data availability

Reasonable data sharing requests may be addressed to the corresponding author in writing (email) and require a formal data sharing agreement. Data sharing agreements must include details on how the data will be stored, who will have access to the data, the intended use of the data, and agreements as to the allocation of intellectual property.

## Results

### CAG repeat number was increased but within the lower pathogenic range

**Fig. 1:**
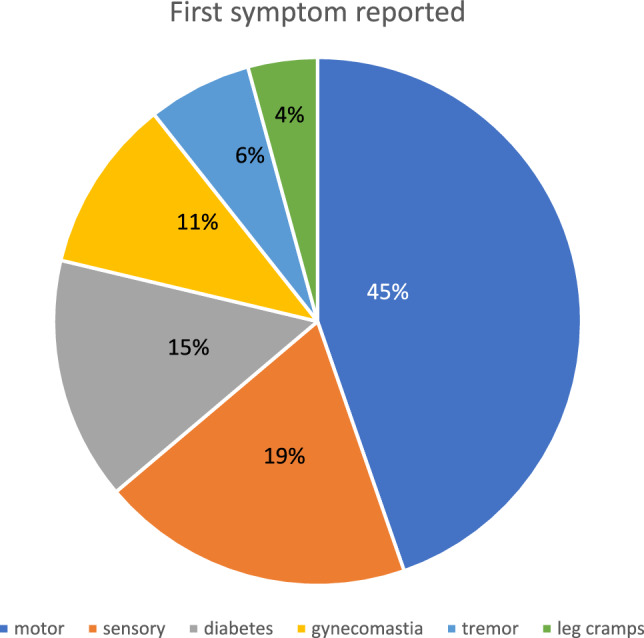
First symptom reported Motor onset was defined as the time of first reported muscular weakness. Motor symptoms were the most common initial presentation, followed by sensory symptoms. Gynecomastia may be underestimated due to incomplete retrospective records. Data on initial symptoms were missing for three individuals, and one individual reported both sensory symptoms and diabetes at onset.

**Fig. 2:**
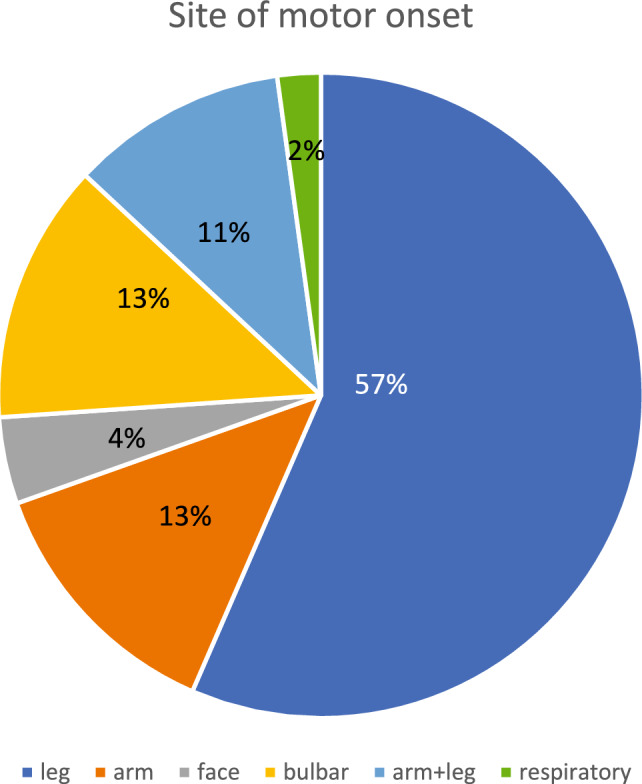
Site of motor onset The most common site of motor onset was the lower extremities, with more than half of the individuals reporting initial leg weakness. Facial onset was defined as initial weakness in facial muscles, resulting in difficulties in chewing or whistling. Bulbar onset was defined as dysphagia, dysarthria or laryngospasm as the initial symptom. Respiratory onset was defined as dyspnea/diaphragm paresis as the first symptom. Information on the site of motor onset was missing for 3 of 49 individuals.

Among the 49 male participants included in our SBMA cohort, 44 originated from northern Sweden, while the remaining five were from the Stockholm region (Central Sweden). The mean number of CAG repeats in the *AR* gene was 43.1 (median 43, range 37–55 repeats). The mean age at onset for all reported symptoms was 52 years (median 53.5, range 18–82), whereas motor symptoms specifically appeared at a mean age of 58.6 years (median 57, range 30–82) (Table 1). Muscular weakness was identified as the initial symptom by approximately 45% of the participants (Fig. 1), with the lower extremities most commonly reported as the initial site of motor involvement (Fig. 2).

**Table 1:** Basic characteristics of the SBMA cohort

Number of individuals	49
Mean age of first nonmotor symptom (years) (*n* = 46)	52.0 ± 12.5 Median 53.5 (range 18–82)
Mean age of first motor symptom (years) (*n* = 46)	58.6 ± 10.3 Median 57 (range 30–82)
Mean number of CAG repeats (*n* = 49)	43.1 ± 2.7 Median 43 (range 37–55)
Mean age at death (years) (*n* = 25)	76 ± 7.6 Median 76.6 (range 52–88)
Mean years with disease (*n* = 23)	20.6 ± 8.7 Median 20 (range 3–37)

Information on age of onset was missing for 3 individuals. 24 individuals were still alive. Five individuals were not from northern Sweden.

Among the 25 deceased individuals, the mean age at death was 76 years (median 76.6, range 52–88), and the mean disease duration was 20.6 years (median 20, range 3–37). Reliable data to estimate disease duration were missing for two individuals. There was one obvious outlier in the study cohort: subject number 49 had 55 CAG repeats, far more than any other participant, and he also had the youngest age of onset (Supplemental Table 1). Interestingly, this patient was born in Central Europe and later moved to Sweden before developing SBMA.

### Lower-range CAG repeat expansions were associated with non-motor symptoms

Overall, 19% of the individuals in the cohort reported sensory disturbances as the initial clinical manifestation of SBMA (Fig. 1), including tingling, coldness, pain, or burning paresthesia. These symptoms frequently occurred several years before the onset of motor deficits. Besides paresthesia, six of nine individuals, reported pain, commonly described as burning feet or abnormal cold sensations. Diabetes mellitus was present in only three of nine individuals, despite follow-up exceeding 20 years. Neurophysiological evidence of neuropathy in most patients suggests that non-diabetic sensory neuropathy was the primary cause of these early symptoms.

Cervical or lumbar pain was reported by 52% of individuals, generally appearing later in the disease course. However, eight individuals reported back pain preceding the appearance of any motor symptoms.

Radiological abnormalities were frequent, with 18 individuals demonstrating spinal alterations on imaging. The most frequently reported radiological abnormalities included disk herniation (*n* = 5); cervical and lumbar spinal stenosis (*n* = 4); and other degenerative changes (*n* = 9), such as spinal canal narrowing (*n* = 3) and spondylolisthesis (*n* = 1). Six individuals underwent spinal surgery.

Regarding androgen-related outcomes, a minority of male participants (7 out of 42 with available data) were childless; of these, three had never cohabitated with a partner (Supplemental Table 1). These findings may reflect varying degrees of androgen insensitivity or the influence of psychosocial factors.

### Cardiovascular comorbidities were prevalent

**Fig. 3:**
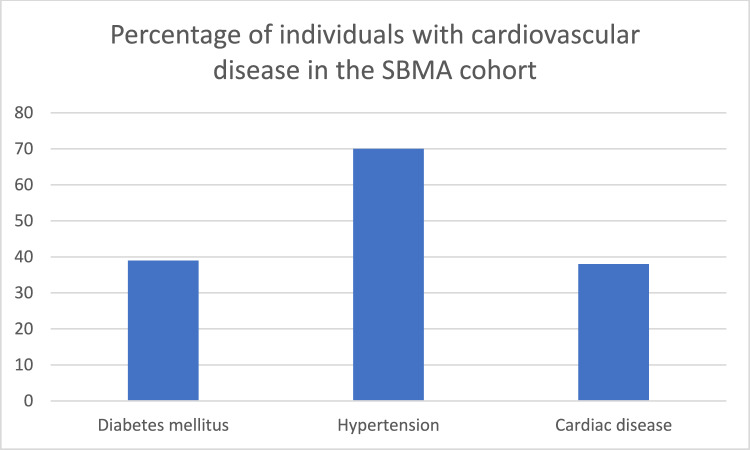
Cardiovascular comorbidities Information on diabetes mellitus, hypertension and cardiac disease was missing for 8, 9 and 7 individuals, respectively. The underlying cardiac diseases were as follows: myocardial infarction (9), angina pectoris (3) (in two cases leading to PCI), cardiac arrest (1), and atrial fibrillation (3) combined with hypertension (1), congestive heart failure (1) or stroke (1) (number of individuals).

**Fig. 4:**
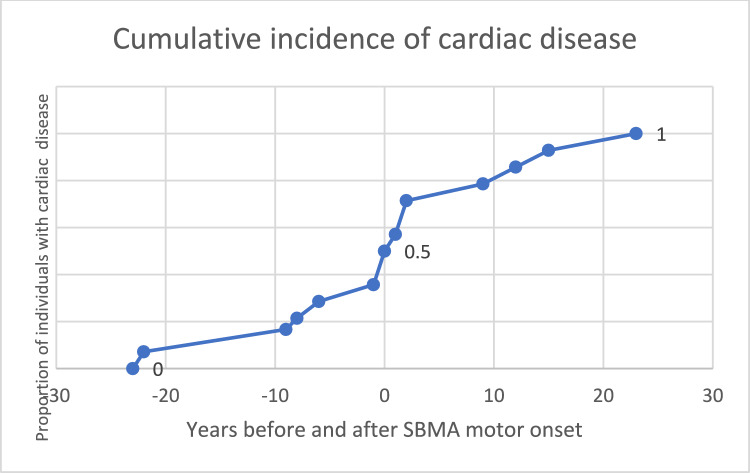
Cumulative incidence of cardiac disease in relation to SBMA motor onset Half of the individuals in the cohort who developed cardiac disease did so prior to SBMA motor onset.

Cardiovascular conditions were common among the participants. Diabetes mellitus was present in 16 of 41 individuals (39%) and hypertension in 28 of 40 (70%) (Fig. 3). Cardiac disease occurred in 16 of 42 individuals (38%), including nine with prior myocardial infarction and three with angina, two of whom underwent percutaneous coronary intervention (PCI). One participant died of sudden cardiac arrest. Additionally, atrial fibrillation was observed in three individuals, all of whom had concomitant comorbidities, including hypertension, hyperlipidemia, prior stroke or congestive heart failure (Supplemental Table 1). No other arrhythmias and no Brugada ECG-pattern was described. In 50% of the individuals, cardiac disease occurred before or at time of motor onset (Fig. 4). The mean age of first cardiac symptom was 64.6 years (Supplemental Table S1).

### Plasma NfL levels correlated with cerebrovascular disease and atypical clinical decline

The prevalence of abnormal muscular and cardiac biomarkers is summarized in Table 2. Elevated CK and myoglobin levels were observed in 29 out of 45 (58%) and 26 out of 45 (64%) individuals, respectively, whereas reduced creatinine was seen in only 20 out of 45 (44%) of individuals. Among the cardiac biomarkers, TnT, TnI and ProBNP levels were elevated in 39 out of 45 (87%), 6 out of 44 (14%) and 6 out of 44 (14%) of participants, respectively. The HbA1c level was elevated in 25% of participants (11 out of 44 individuals), nine of whom had no diabetes mellitus diagnosis.

**Table 2:** Muscular and cardiac biomarkers

Biochemical profile	SBMA mean ± SE (range)	Reference range	Below reference range (%)	Above reference range (%)
Myoglobin (µg/l)	141,3 ± 94,2 (29,5–428)	28–72	0	**64%**
ProBNP (ng/l)	107,9 ± 120,7 (15,4–467)	< 75 years: < 100 > 75 years: < 400	0	**14%**
TnT (ng/l)	46,2 ± 54,8 (7,2–320)	< 14,51	0	**87%**
TnI (ng/l)	22,3 ± 20,7 (5–58) ^*†*^	< 5	0	**14%**
CK (µkat/l)	8,0 ± 6,7 (1,3–30,4)	18–50 years: < 6,7 > 50 years: < 4,7	0	**58%**
Creatinine (µmol/l)	64,2 ± 20,9 (22,1–128)	60–105	**44%**	**4%**
HbA1c (mmol/mol)	43,2 ± 12,3 (32,0–95,5)	< 46	0%	**25%**

Information was missing for four individuals in the cohort. Analytical data were collected from 45 individuals, except for ProBNP, HbA1c and TnI (n = 44). †: Estimates of the mean and range were made from six elevated values. The remaining 38 samples were within the normal range (< 5 ng/l)

**Fig. 5:**
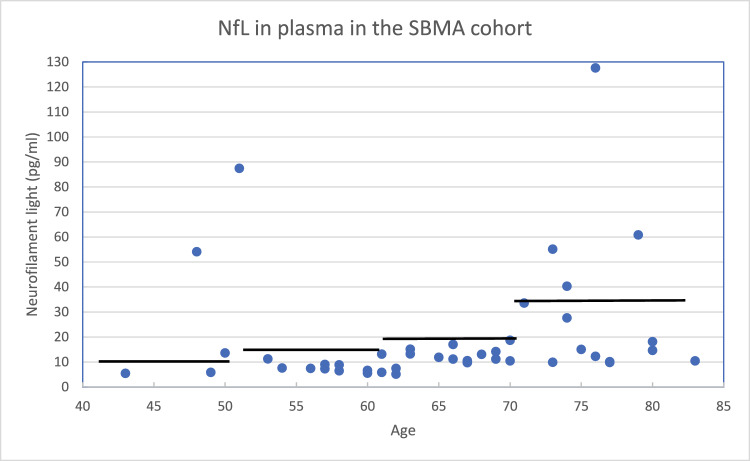
Neurofilament light chain in plasma Plasma NfL levels in the SBMA cohort were age dependent, and the solid black lines represent the cutoff for each age category, according to Simren et al. [9]. Among the 45 individuals tested, seven had elevated pNfL levels. Two values were slightly above the age-specific reference value. The individual with the greatest age-adjusted pNfL elevation had a *C9ORF72* hexanucleotide repeat expansion.

**Fig. 6:**
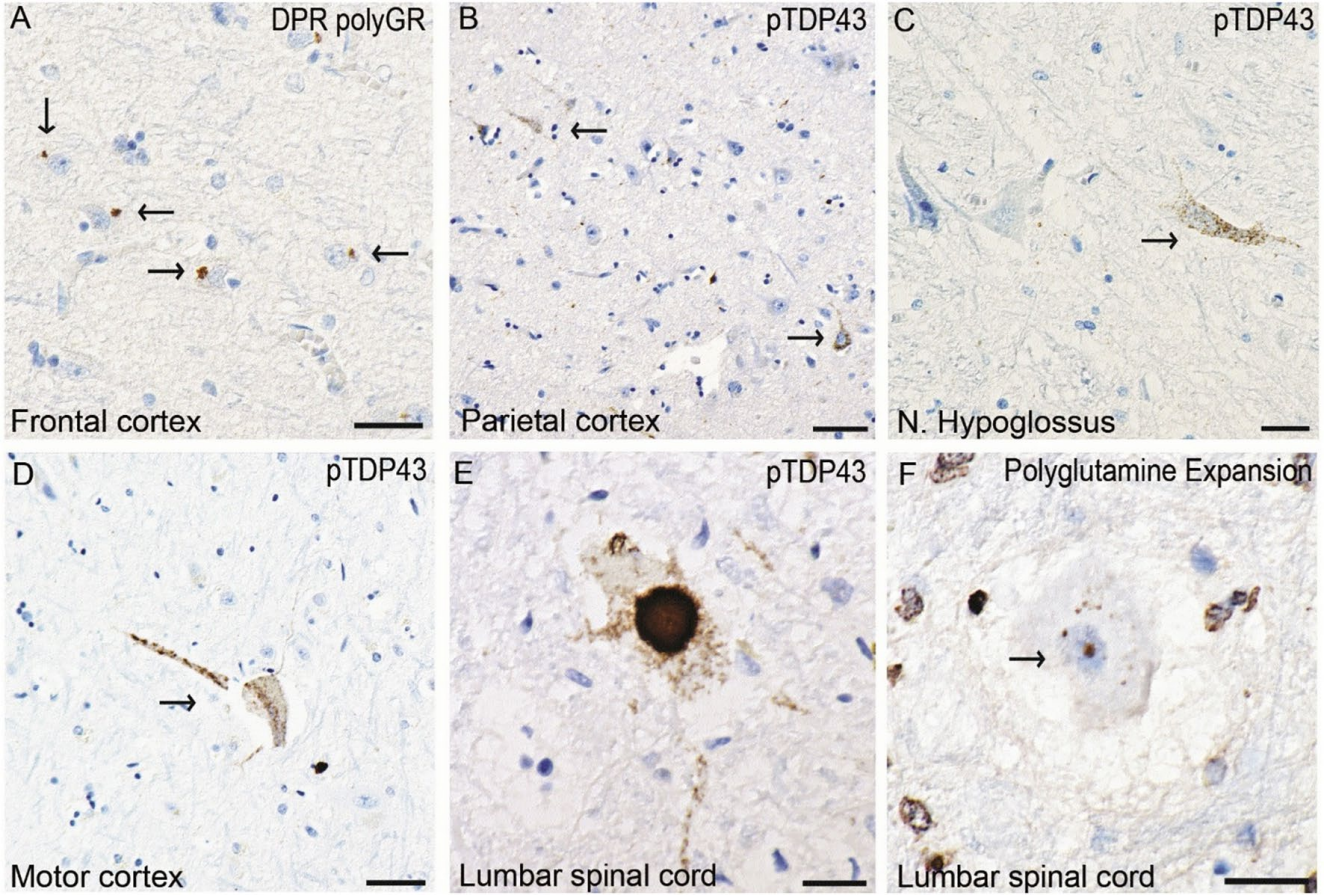
pTDP-43 inclusions in two SBMA patients Images represent patient 2 in **A-C** and patient 21 in **D-F**. (**A**) depicts cytoplasmic DPR poly-GR inclusions in the frontal cortex. (**B**) illustrates granular cytoplasmic pTDP-43 inclusions in neurons in the parietal cortex. The same type of pTDP-43 inclusion is also seen in the hypoglossal nucleus in (**C**). (**D**) depicts pTDP-43 inclusions in a Betz cell in the motor cortex, and (**E**) depicts pTDP-43 inclusion in a lumbar motor neuron. In (**F**), granular polyQ inclusions are depicted in the nucleus of a motor neuron of the lumbar spinal cord stained with an antibody against polyglutamine expansions. The scale bars represent 50 µm in **A-B**; 30 µm in **D**; and 20 µm in **C**, **E** and **F**.

PNfL levels, presented in Fig. 5, were elevated in seven out of 45 individuals. Among those with elevated pNfL levels, five had diabetes or impaired glucose tolerance, four had cardiac disease, and three had ischemic white matter lesions or stroke. Two individuals with elevated pNfL levels underwent autopsy. One patient (Supplemental Table 1, No. 2) also harbored a *C9ORF72* hexanucleotide repeat expansion (HRE). In this patient, dipeptide repeat (DPR) inclusions were identified in the frontal cortex (Fig. 6a), and phosphorylated granular TDP-43 (pTDP-43) was detected in the parietal cortex (Fig. 6b), in the hypoglossal nucleus (Fig. 6c) and at all levels of the spinal cord. Notably, no intranuclear polyQ inclusions were detected in this individual; however, only limited material was available. The second autopsied individual (Supplemental Table 1, No. 21) exhibited extensive pTDP-43 cytoplasmic inclusions in the motor cortex (Fig. 6d), the hypoglossal nucleus, and motor neurons across all spinal cord levels (Fig. 6e) as well as intranuclear polyQ inclusions within spinal motor neurons (Fig. 6f). Taken together, the neuropathological findings in these two individuals correspond to a histopathological diagnosis of ALS in addition to the existing SBMA diagnosis.

## Discussion

We here describe a mainly northern Swedish SBMA cohort comprising 49 individuals, characterized by a lower average CAG repeat length than in previous international studies, a later age of motor symptom onset, and a greater frequency of sensory symptoms preceding motor involvement. Additionally, cardiovascular disease and diabetes mellitus were commonly observed comorbidities and were correlated with biomarkers.

The mean CAG repeat length in our cohort was 43.1, which is lower than the global average reported for SBMA patients (46.5 repeats) [1] but consistent with previous findings from other Nordic SBMA cohorts, where the reported mean CAG repeat length was 43.4–43.5 [10–12]. Interestingly, our cohort had a delayed onset of motor symptoms, with a mean age of onset of 58.6 years—approximately fifteen years later than what has been reported in other SBMA populations [1]. These findings confirm previous reports of an inverse correlation between CAG repeat length and both age at motor onset [13–15] and muscle strength and function [13,16]. Consistent with Atsuta et al. [17], repeat length did not appear to affect the rate of disease progression in our cohort.

Overall, most men in this cohort presented with nonmotor symptoms (Fig. 1). Leg pain or paresthesia was the most frequent initial nonmotor symptom, often preceding motor onset by several years. Although sensory symptoms have been reported in SBMA [18,19], they are rarely regarded as early or prominent features [20]. Nonetheless, our findings align with those of previous reports, suggesting an association between lower CAG repeat length (< 47) and a predominantly sensory SBMA phenotype [21,22]. In addition to sensory onset symptoms, back pain was frequently described before other SBMA symptoms and was reported in more than half of the individuals overall (Supplemental Table 1), which is worth investigating further in future studies of SBMA cohorts.

A notably high prevalence of diabetes mellitus was observed in this SBMA cohort in northern Sweden (Fig. 3). It was a common first symptom and was present in 39% of individuals. An additional 18% had elevated HbA1c levels, indicating impaired glucose tolerance. A worldwide population study (MONICA) conducted in 1985–2004 highlighted a high prevalence of cardiovascular disease in the general population in northern Sweden [23]. Here, the prevalence of diabetes mellitus among men aged 65 years or older was reported to be 15.3% in 2009 [24]. Consequently, the prevalence of diabetes mellitus in our SBMA cohort was more than 2.5-fold greater than the age-specific average in Sweden. While diabetes is a known comorbidity of SBMA [25], cardiac disease associated with SBMA is rarely reported in the literature [20]. A comparative summary of cardiovascular findings from previous cohort studies is provided in Supplementary Table S4. Two other SBMA cohorts—characterized by higher mean CAG repeat lengths (46.0 and 45.9, respectively)—reported low or zero rates of ischemic heart disease [26,27]. A study from Japan reported a high prevalence of ECG abnormalities, but seldom a history of cardiovascular disease [28]. In our cohort, cardiac disease was reported in 38% of participants (Fig. 3). In half of these cases, cardiac symptoms developed before or at the time of SBMA motor onset (Fig. 4; age of onset details are provided in Supplementary Table S1). Estimating the expected incidence and prevalence of myocardial infarction (MI) is challenging because of declining event rates over recent decades. In the population of northern Sweden, according to the aforementioned MONICA study, the annual incidence of MI among males aged ≥ 55 years was approximately 600 per 100,000 during the period of 1994–2004 [29] and 400 per 100,000 among men aged 45–74 years in Sweden in 2023 [30]. Based on the mean age and year of inclusion in our cohort, this corresponds to an expected cumulative MI risk of approximately 7–11%. However, 18% of our cohort had a documented history of MI—a markedly higher incidence than anticipated, and one that has not previously been reported in any SBMA population. To further investigate cardiac involvement, we analyzed serum levels of cardiac biomarkers in stored samples. In our cohort, 86% of individuals had elevated TnT values. While TnT can be elevated because of skeletal muscle damage [31] or comorbid conditions such as diabetes mellitus or renal impairment [32], its interpretation in SBMA remains challenging. TnI, which is considered more specific to myocardial injury [32], was elevated in 14% of individuals—significantly higher than previously reported in SBMA cohorts [31] (Table 2). Elevated TnI in the absence of acute symptoms is indicative of chronic myocardial injury and is predictive of cardiovascular mortality [32]. When stratifying individuals by CAG repeat size—comparing those with lower and higher repeat than the cohort mean—we found no association between cardiac disease and CAG repeat number (p-value 0.95), see Supplemental Table 5. However, all individuals except two within the cohort had a repeat size less than mean when comparing with international studies [1], so we cannot exclude that CAG repeat size influence cardiovascular risk. The high prevalence of metabolic and cardiovascular symptoms in this cohort—particularly among individuals with shorter CAG repeat lengths—suggests a heightened risk of developing a distinct metabolic or cardiovascular phenotype within this patient population. The discrepancies in the prevalence of diabetes mellitus and cardiovascular disease in individuals with SBMA across studies may also reflect differences in age of study population, genetic background, environmental factors, or methodology but underscore the need for further investigations of the cardiovascular–metabolic risk profile in carriers of all sizes of CAG repeat expansions in the *AR gene*, including female carriers.

Most patients in our cohort had normal pNfL levels, which is consistent with the findings of previous studies [33,34]. However, seven individuals had elevated pNfL levels (Fig. 5), raising questions about the underlying pathophysiological mechanisms. The elevated pNfL levels ranged from slightly above the age-adjusted reference values in two individuals to sixfold higher. Three individuals with elevated pNfL levels all had evidence of cerebrovascular disease in terms of history of ischemic encephalopathy, a recent stroke or extensive white matter lesions, in addition to diabetes accompanied by myocardial infarction or heart failure. Diabetes mellitus [35], myocardial infarction [36], and stroke [37] are all correlated to increased pNfL levels. Elevated pNfL levels may thus reflect the high cerebro- and cardiovascular disease burden in these patients. The individual with the greatest age-adjusted increase in pNfL was also found to harbor a *C9ORF72* hexanucleotide repeat expansion (Supplemental Table 1, subject No. 2), as confirmed by a Southern blot. He developed both upper and lower motor neuron signs and died after a three-year disease course. Blood leukocyte DNA analysis revealed 38 CAG repeats in the *AR* gene, but postmortem studies revealed DPR inclusions as well as pTDP-43 inclusions in both upper and lower motor neurons (Fig. 6), supporting *C9ORF72HRE*-related ALS. No intranuclear polyQ-inclusions were found, but only limited tissue samples were available for analysis. Collectively, these findings suggest that his phenotype was more consistent with *C9ORF72HRE*-induced ALS than with classical SBMA and align with the elevated pNfL concentration [38]. Patient No. 21 (Supplementary Table 1), with 42 CAG repeats in the *AR* gene and with elevated pNfL levels, also exhibited a more rapid clinical decline than typically observed in SBMA, dying of aspiration pneumonia after a three-year symptomatic disease course. Postmortem examination revealed classical intranuclear polyQ inclusions, most likely representing AR inclusions and consistent with SBMA pathology, but also showed pTDP-43-positive inclusions in the motor cortex, hypoglossal nucleus and spinal cord (Fig. 6), findings not typically seen in SBMA [39]. These observations support a concomitant ALS and SBMA diagnosis, where the ALS of unknown etiology likely represents the primary driver of the phenotype and progression in this atypical case. The elevated pNfL levels observed in these two individuals underscore the potential of pNfL as a biomarker for atypical or accelerated disease trajectories in cases deviating from the classical SBMA phenotype. Additionally, one can hypothesize that *AR* gene expansions may, in themselves, confer an increased risk of ALS—analogous to associations previously reported for intermediate CAG repeat expansions in two other genes, *ATXN2* [40] and *HTT* [41], except that larger expansions in these genes are primarily associated with other phenotypes (SCA2 and Huntington’s disease, respectively). Further neuropathological studies are needed to clarify these findings.

## Limitations

This study has several limitations. As most clinical data were collected retrospectively, some key information was missing or incomplete. Clinical data were missing for three individuals, and comorbidity data were incomplete for several deceased individuals. The unusually high cardiovascular burden found in the study may at least partly reflect the older age of this cohort. Additionally, symptoms that were less disruptive to everyday life—such as gynecomastia—may have gone unrecorded, particularly in neurology records. Similarly, assessing infertility or reduced fertility retrospectively was challenging because of limited documentation. Several patients were referred to the neurology department only because the general practitioner or other referring physician suspected ALS; therefore, we speculate that SBMA patients who do not develop the full paretic phenotype may never be referred to a neurologist and may not receive a diagnosis of SBMA. Finally, as 49% of individuals in the study cohort are still alive, the presented estimation of disease duration is based on a subset of our cases.

## Conclusion

In this Swedish SBMA cohort, we observed a lower average CAG repeat length and a later age of motor onset than in previously reported international cohorts [1]. Many individuals initially presented with nonmotor symptoms, such as cardiovascular disease, sensory symptoms, and back pain, often years before the onset of fasciculations and later motor paresis and atrophy. These findings align with those of a recent study reporting a tenfold higher frequency of repeat expansions in the *AR* gene in European and North American populations than previously estimated, suggesting that SBMA may be underdiagnosed because of its pleomorphic clinical presentation^42^. Our results emphasize the need to broaden the clinical definition of SBMA to include sensory, metabolic and cardiovascular features. Importantly, the high prevalence of diabetes mellitus and ischemic heart disease in our cohort indicates underrecognized cardiac vulnerability in SBMA patients and highlights the necessity of routine screening for these comorbidities. Elevated pNfL was observed only in individuals with extensive cerebrovascular or cardiovascular disease, or with atypical and rapidly progressive clinical decline, and should serve as a red flag to investigate comorbidities and/or alternative diagnoses.

## Supplementary Information

Below is the link to the electronic supplementary material.

Supplementary file1 (DOCX 22 KB)

Supplementary file2 (PDF 19 KB)

Supplementary file3 (PDF 34 KB)

Supplementary file4 (DOCX 19 KB)

Supplementary file5 (DOCX 15 KB)
