# Supplementary material for: Heterogeneous phenotype and cardiovascular comorbidities in Swedish patients with spinobulbar muscular atrophy
Source: J Neurol. 2026 Jan 10;273(1):75. doi: 10.1007/s00415-025-13605-z (PMC12789218; doi:10.1007/s00415-025-13605-z)
Supplement: Supplementary file 1 — Supplementary file1 (DOCX 22 KB) [file 415_2025_13605_MOESM1_ESM.docx]

**Supplemental Table S1. Clinical information of individuals in the SBMA cohort.**

| Pat. No. | First symptom | First motor symptom | Comorbidity  (age in years) | Number of children | CAG repeat length |
| --- | --- | --- | --- | --- | --- |
| 1 | Sensory (feet) | Leg | Diabetes mellitus. Angina, CABG (68). Myocardial infarction (76). Back pain. L3-L4 spondylosis with disc degeneration. | 2 | 37 |
| 2 | Arm | Arm | *C9ORF72* hexanucleotide repeat expansion. | - | 38 |
| 3 | Diabetes | Leg | Diabetes mellitus. Myocardial infarction and hypertension (56). Pain in left leg. L4-L5 disc herniation surgery. | 2 | 40 |
| 4 | Gynecomastia | Leg | Hypertension. | 1 | 40 |
| 5 | Dyspnea | Dyspnea | Asthma. Hypertension. Hyperlipidemia. Restless legs. | 3 | 40 |
| 6 | Arms and legs | Arms and legs | - | - | 41 |
| 7 | Sensory (leg + arms) | Leg | Diabetes mellitus. Hypertension. Hyperlipidemia. | 3 | 41 |
| 8 | Gynecomastia | Leg | Hypertension. Unstable angina (76). PCI. Congenital narrow spinal canal. Foraminal cervical stenoses. | 2 | 41 |
| 9 | Muscle cramps | Leg | Thoracic compression, trauma. | 3 | 41 |
| 10 | Diabetes | Leg | Diabetes mellitus. Myocardial infarction (47). Hypertension. Hyperlipidemia. CABG. L5-S1 spondylolisthesis. | 4 | 41 |
| 11 | Leg cramps | Arm | Infertility, adopted children. | 0 | 41 |
| 12 | Gynecomastia | Bulbar | Lower back pain. Myocardial infarction (70). Hypertension. Hyperlipidemia. RCA stent due to chest pain. Prostate cancer. | 2 | 41 |
| 13 | Diabetes | Arm+ leg | Diabetes mellitus. Hypertension. Back pain after trauma (compression of L1).  No children, no partner. | 0 | 42 |
| 14 | Arm | Arm | Hypertension. Hyperlipidemia. Diabetes mellitus. Neck pain. Cervical spinal stenosis, surgery. | 2 | 42 |
| 15 | - | - | Hypertension. Hyperlipidemia. | - | 42 |
| 16 | Leg | Leg | No information. | 3 | 42 |
| 17 | Leg | leg | Hypertension. Back pain. | 2 | 42 |
| 18 | Atrophy leg | Leg | Back pain. | 2 | 42 |
| 19 | Sensory (arm) | Arm | Hypertension. Neck pain. | 2 | 42 |
| 20 | Bulbar | Bulbar |  | 2 | 42 |
| 21 | Bulbar | Bulbar | Penile cancer. Hip fracture. Hypothyroidism. | 2 | 42 |
| 22 | Leg | Leg | Hypertension.  Myocardial ischemia (81). Intermittent claudication. Extensive L3-L4 and L5-S1 lumbar spondylosis. | 1 | 43 |
| 23 | Sensory (leg) | Leg | No children. Hypertension. | 0 | 43 |
| 24 | Leg | Leg | Hypertension. MR findings: narrow cervical spinal canal but no spinal stenosis. No children. | 0 | 43 |
| 25 | Face | Face | Hypertension. Diabetes mellitus. | 3 | 43 |
| 26 | Diabetes | Leg | Diabetes mellitus. Atrial fibrillation (N/A). Hypertension. Hyperlipidemia. | 2 | 43 |
| 27 | Leg | Leg | Hypertension. Diabetes mellitus. Cardiac arrest (69). No children. | 0 | 43 |
| 28 | Diabetes | Arm+leg | Diabetes mellitus. Hypertension. Hyperlipidemia. Myocardial ischemia × 3 (57). PCI. Back pain. Disc herniation surgery. | 2 | 43 |
| 29 | Sensory (arm), diabetes | Face | Hypertension. Hyperlipidemia. Stroke. Myocardial infarction (55). Diabetes mellitus. | 3 | 43 |
| 30 | Sensory (leg) | Leg | Hypertension. Angina pectoris (60). Myocardial ischemia (72). L4-L5 disc herniation. Cervical spondylosis. | 3 | 44 |
| 31 | Leg | Leg | Diabetes mellitus. Back pain. | 4 | 44 |
| 32 | Gynecomastia | Arm + leg | Back pain. Cervical disc herniation, surgery. Lumbar disc herniation. | 2 | 44 |
| 33 | Arm | Arm | Back pain and shoulder pain. Cervical and lumbar spinal stenosis with cervical laminectomy. | 1 | 44 |
| 34 | Sensory (leg) | Leg | Hypertension. Angina pectoris. Repeated PTCA. Stent (75). Disc herniations. Back pain after trauma. | 3 | 44 |
| 35 | Leg | Leg | No information. | - | 44 |
| 36 | Gynecomastia | Arm+ leg | No children, no partner. | 0 | 44 |
| 37 | Tremor (arm) | Arm | Whiplash trauma. Disc degeneration. | 2 | 44 |
| 38 | Diabetes † | Leg† | Seminoma. Myasthenia gravis. Diabetes mellitus. Hypertension. Hyperlipidemia. Heart failure and atrial fibrillation (72). Stroke. | 2 | 44 |
| 39 | Face | Face | Diabetes mellitus. Neck pain. | 1 | 45 |
| 40 | - | - | No information. | - | 45 |
| 41 | - | - | No information. | - | 45 |
| 42 | Leg | Leg | Angina pectoris (62). Hypertension. No children, no partner. | 0 | 45 |
| 43 | Sensory (leg), leg cramps | Face | C4-C7 foraminal stenosis. | 3 | 45 |
| 44 | Sensory (leg) | Leg | Atrial fibrillation (56). Stroke. Hyperlipidemia. Hypertension. Lumbar pain. C6-root compression. | 2 | 46 |
| 45 | Leg | Leg | Hypertension. | 2 | 46 |
| 46 | Tremor (arms) | Leg | Diabetes mellitus. Hypertension. Hyperlipidemia. | 3 | 46 |
| 47 | Leg | Leg | Breast reduction surgery. | 2 | 46 |
| 48 | Tremor (arms) | Leg | Lower back pain. Lumbar spinal stenosis surgery. Hypertension. | 1 | 47 |
| 49 | Legs | Legs | Myocardial infarction (N/A). Diabetes mellitus. | - | 55 |

*Individuals are described in order from shortest to longest repeat length. –: missing clinical data. †: time of first symptom hard to estimate owing to myasthenia gravis. N/A: not available.*
