# Supplementary material for: Heterogeneous phenotype and cardiovascular comorbidities in Swedish patients with spinobulbar muscular atrophy
Source: J Neurol. 2026 Jan 10;273(1):75. doi: 10.1007/s00415-025-13605-z (PMC12789218; doi:10.1007/s00415-025-13605-z)
Supplement: Supplementary file 2 — Supplementary file2 (PDF 19 KB) [file 415_2025_13605_MOESM2_ESM.pdf]

Supplemental Table S2. Primers for AR fragment amplification.

SBMA Fw	FAM-5’-TCCAGAATCTGTTCCAGAGCGTGC-3’
SBMA Rev	5’-GCTGTGAAGTTGCTGTTCCTCAT-3’
