# Supplementary material for: Heterogeneous phenotype and cardiovascular comorbidities in Swedish patients with spinobulbar muscular atrophy
Source: J Neurol. 2026 Jan 10;273(1):75. doi: 10.1007/s00415-025-13605-z (PMC12789218; doi:10.1007/s00415-025-13605-z)
Supplement: Supplementary file 3 — Supplementary file3 (PDF 34 KB) [file 415_2025_13605_MOESM3_ESM.pdf]

Supplemental Table S3. PCR conditions for AR fragment amplification.

94°C	2 min					
94°C	30 s	58-53°C (-1°C/cycle	1 min	68°C	1 min	6 cycles
94°C	30 s	52°C	1 min	68°C	1 min	25 cycles
68°C	5 min					
10°C	∞					
