# Supplementary material for: Heterogeneous phenotype and cardiovascular comorbidities in Swedish patients with spinobulbar muscular atrophy
Source: J Neurol. 2026 Jan 10;273(1):75. doi: 10.1007/s00415-025-13605-z (PMC12789218; doi:10.1007/s00415-025-13605-z)
Supplement: Supplementary file 4 — Supplementary file4 (DOCX 19 KB) [file 415_2025_13605_MOESM4_ESM.docx]

***Supplemental Table 4. Comparative table summarizing findings from previous cohort studies.***

| **Author/ Journal/**  **doi-number** | **Number of individuals**  **in cohort** | **Mean age of onset in years**  **(years at evaluation)** | **Mean number of CAG repeats** | **Sensory deficits/ tingling**  **(%)** | **Hyper-cholesterolemia/ hyper-triglyceridemia**  **(%)** | **Diabetes mellitus**  **(%)** | **Hyper-tension (%)** | **Cardiac disease**  **(%)** |
| --- | --- | --- | --- | --- | --- | --- | --- | --- |
| Ni et al. Plos One 2015  doi: 10.1371/journal. pone.0122279 | 155 | 44.2 | 48.6 | - | 22/66  Measurements from  29 and 27 individuals respectively | **-** | **-** | **-** |
| Araki et al. Neurology 2014.  doi:10.1212/WNL.0000000000000434 | 144 | 43.5  (52.1) | 48.1 | _ | 41.7/ 49.3 | _ | 37.6 | 7.6  (Angina in 4 cases, myocardial infarction, aortic regurgitation, bradycardia, pulmonary artery stenosis with ventricular septal defect, arrhythmia, cardiac hypertrophy, or aortic dissection in one case each)  ECG-abnormalities in 48.6%,  whereof Brugada-type changes in 11.8% |
| Rosenbohm et al. Journal of Neurology 2018  doi: 10.1007/s00415-018-8790-2 | 80 | 44.4  (55.9) | 46.2 | 64 | 78/56 | 11,3  (28% elevated fasting blood sugar level) | - | - |
| Querin et al.  JNNP, 2016  doi:10.1136/ jnnp-2015-311305 | 73 | 42.3  (57.3) | 45.9 | - | 54.7/48 | -  (41 % elevated fasting blood sugar level) | 37 | 4 ischemic heart disease  4 Brugada-like ECG changes |
| Rhodes et al. Brain 2009  doi:10.1093/brain/awp258 | 57 | 41  (53) | 46.7 | - | 53/- | -  (12 % elevated fasting blood sugar level) |  | ­- |
| Francini et al. Acta Myologica 2018.  PMCID: PMC6390113 | 47 | -  (57.7) | - | - | 51/38 | -  (49% elevated fasting blood sugar level) | - | - |
| Mariotto et al. Neuromuscular Disorders 2000  doi: 10.1016/s0960-8966(99)00132-7 | 36 | 46.2  (55.7) | 44.6 | 55 | - | - | - | - |
| Dejager et al. J Clin Endocrinol Metab, 2002  doi: 10.1210/jcem.87.8.8780 | 22 | 42.8  (50.5) | 46 | - | 68/41 | - | 136 | No coronary heart disease |
| Sinnreich et al. Can J Neurol Science 2004    doi:[10.1017/S0317167100003486](https://doi.org/10.1017/S0317167100003486) | 20 | 43.45 | 46.25 | -  (55% distal sensory loss on examination) | - | 30  (+ 15% with glucose impairment) | - | - |
| Nishiyama et al. J of Clinical Neuroscience 2014  doi: 10.1016/j.jocn.2013.07.026 | 12 | 45.3  (50.8) | 47.25 | 16.7 | - | - | - | - |
| Roos et al (this cohort) | 49 | 58.6 | 43.1 | -  (19% as first symptom) | - | 39  (+18% elevated HbA1c) | 70 | 38  Myocardial infarction 9/42  Angina pectoris 3/42  Cardiac arrest 1/42  Atrial fibrillation 3/42 |

.
