# Supplementary material for: Heterogeneous phenotype and cardiovascular comorbidities in Swedish patients with spinobulbar muscular atrophy
Source: J Neurol. 2026 Jan 10;273(1):75. doi: 10.1007/s00415-025-13605-z (PMC12789218; doi:10.1007/s00415-025-13605-z)
Supplement: Supplementary file 5 — Supplementary file5 (DOCX 15 KB) [file 415_2025_13605_MOESM5_ESM.docx]

***Supplemental Table S5. Cardiac disease and sensory onset in relation to CAG repeat number.***

| CAG repeat group | Cardiac disease | No cardiac disease | p-value | Sensory onset | No sensory onset | p-value |
| --- | --- | --- | --- | --- | --- | --- |
|  |  |  |  |  |  |  |
| <44 repeats | 10 | 16 | 0.95 | 5 | 23 | 0.72 |
| 44 or> repeats | 6 | 10 |  | 4 | 14 |  |
|  |  |  |  |  |  |  |
|  |  |  |  |  |  |  |

Data was missing regarding cardiac disease for 7 individuals and regarding onset symptoms for 3 individuals.
